# Daily stressor exposure and subjective memory: Age moderation and within-person variation across days and years

**DOI:** 10.1093/geroni/igaf122.3743

**Published:** 2025-12-31

**Authors:** Nicole Stuart, Nancy Sin

**Affiliations:** University of British Columbia, Vancouver, British Columbia, Canada; University of British Columbia, Vancouver, British Columbia, Canada

## Abstract

Growing research indicates that daily stress is associated with poor same-day memory performance. However, it is unclear how these associations may unfold over time, and how age may moderate this relationship. In this pre-registered study, ecological momentary assessment data were collected from adults aged 25-89 (M = 46.23, SD = 17.44 at baseline) across British Columbia, Canada. Annually for 3 years, participants (N = 169; 70% women) reported daily stressor exposure and subjective memory performance for 14 days. Multilevel models evaluated daily stressor exposure and an Age x Yearly Stressor Exposure interaction as predictors of subjective memory, controlling for demographics and depressive symptoms. Subjective memory was rated as relatively worse on days when at least one stressor occurred, compared to stressor-free days (b = -0.126, CI[-0.044,-0.199]). Participants also reported poorer average memory during years with a higher proportion of stressor days, compared to years with fewer stressor days (b = -0.092, CI[-0.064,-0.118]). Baseline age moderated this relationship, showing the strongest deleterious associations in younger (25-39y) adults; however, this relationship remained significant across age groups. Extending prior naturalistic research, the present findings suggest that more stressful periods in one’s life are associated with poorer subjective memory, and this is particularly pronounced among younger adults. Future work should evaluate potential mechanisms underlying this relationship, including age-related differences in stressor qualities, emotion regulation, perseverative cognitive processes, and coping.

